# Thromboembolic risks in patients with COVID-19: major concern to consider in our management

**DOI:** 10.11604/pamj.2020.35.2.22945

**Published:** 2020-04-27

**Authors:** Mazou Ngou Temgoua, Liliane Mfeukeu Kuaté, William Ngatchou, Aurelie Sibetcheu, Zouliatou Nzina Toupendi, Grace Belobo, Alice Ossa, Samuel Kingue

**Affiliations:** 1 Faculty of Medicine and Biomedical Sciences, Department of Medicine and Specialities, Yaoundé, Cameroon; 2 Faculty of Medicine and Pharmaceutical Sciences, Department of Surgery, Douala, Cameroon; 3 Faculty of Medicine and Biomedical Sciences, Department of Pediatrics, Yaoundé, Cameroon; 4 Faculty of Medicine and Biomedical Sciences, Department of Radiology and Medical Imaging, Yaoundé, Cameroon

**Keywords:** COVID-19, Thromboembolism risks, management

## Abstract

COVID-19 pandemic is an emergent cardiovascular risk factor and a major cause of mortality worldwide. Thromboembolism is highly suspected as a leading cause of death in these patients through vascular inflammation caused by SARS COV2. Until now there is no real treatment of COVID-19 and many proposed drugs are under clinical trials. Considering the high incidence of thromboembolic events in critically ill patients with COVID-19, prevention of this disorder should be essential in order to reduce mortality in these patients.

## To the editors of Pan African Medical Journal

After Severe Acute Respiratory Syndrome Coronavirus (SARS COV) in 2003 and Middle East Respiratory Syndrome (MERS COV) in 2012, the new Coronavirus SARS COV2 is a leading infectious cause of mortality worldwide. Originating from Wuhan in China, the disease rapidely spread to 143 countries by March 2020 and is responsible for healthcare resources depletion [1]. The disease is transmitted mainly through interhuman contact through droplets, other routes of transmission like blood, urine or faeces could also be incriminated. The virus invades alveoli through spike protein(S) which links to the Angiotensin Coverting Enzyme 2 (ACE2). Essential factors that determine the virulence of the pathology are: advanced age, hypertension, diabetes, obesity, cancer, pregnancy and chronic kidney disease [2]. The overall crude case fatality ratio is around 1.38%, but this proportion is more high in risk groups particularly in patients aged 80 years or older where the case fatality rate may reach 18.4%. Patients died mainly because of acute respiratory distress syndrome or septic shock [3].

Recently the role of thromboembolic events has been suspected. In a study done by Cui et al. who enrolled 81 patients in Intensive Care Unit (ICU) in China, the incidence of venous thromboembolism (VTE) in these patients was 25% with a death rate of 9.8% [4]. Klok et al. found almost the same incidence in Holland, in a total of 184 patients admitted in ICU, 27% developed thromboembolic complications [5]. The potential mechanisms associated with thromboembolism in COVID-19 could be excessive inflammation, hypoxia, immobilisation and diffuse intravascular coagulation [5]. The risk may be enhanced in patients with known thromboembolic risk factors like old age, obesity, cancer, pregnancy and could partially explain the susceptibility of death in this population [3]. Tang et al. in China also found that high levels of D-dimers were linked to poor prognosis of patients and that prophylactic use of heparin for at least 7 days significantly reduced death [6]. Patients with COVID-19 can rapidely develop severe complications such as renal and pulmonary failure, liver dysfunction which can affect both VTE and bleeding status [2,7].

Since COVID-19 patients with high risk of VTE had poor outcomes compared to those with low risk, the thromboembolism risk should be accurately evaluated [8]. Many comprehensive scores such as Caprini, Padua had been proposed for the VTE and bleeding risk assesment in various clinical settings [7-9]. Given the increase risk of thromboembolism in COVID-19 patients particularly those in ICU, it is important to implement prophylactic use of anticoagulation and/or mechanical compression stockings in order to prevent death of these patients. Unfortunately, a recent study in China showed that this recommandation is not always respected [8]. It should also be noted that there is a corresponding increase in the risk of bleeding due to the sepsis in these patients. Tang et al. found that COVID-19 patients who died in ICU have longer prothrombin time and activated partial thromboplastin time [10]. So it is also very important to assess the risk of bleeding in patients before the use of anticoagulation.

Thromboembolism is highly prevalent in COVID-19 patients and contributes as an important cause of death. Prophylactic measures to prevent it’s incidence should be implemented in all protocols however bearing in mind the risk of bleeding.

## Competing interests

The authors declare no competing interests.
